# Disparities in Confidence to Manage Chronic Diseases in Men

**DOI:** 10.3934/publichealth.2014.3.123

**Published:** 2014-08-04

**Authors:** Keith Elder, Keon Gilbert, Louise Meret Hanke, Caress Dean, Shahida Rice, Marquisha Johns, Crystal Piper, Jacqueline Wiltshire, Tondra Moore, Jing Wang

**Affiliations:** 1 College for Public Health and Social Justice, Department of Health Management and Policy, Saint Louis University, Saint Louis, MO 63103, USA; 2 College of Health and Human Sciences, Department of Public Health Sciences, University of North Carolina Charlotte, Charlotte, NC 28223, USA; 3 College of Public Health, Department of Health Management and Policy, University of South Florida, Tampa, FL 33620, USA; 4 College of Pharmacy and Health Sciences, Department of Health Sciences, Texas Southern University, Houston, TX 77004, USA

**Keywords:** men, health information, confidence, self-management, chronic illnesses

## Abstract

**Background:**

Chronic diseases are highly prevalent among men in the United States and chronic disease management is problematic for men, particularly for racial and ethnic minority men.

**Objectives:**

This study examined the association between health information seeking and confidence to manage chronic diseases among men.

**Methods:**

Study data were drawn from the 2007 Health Tracking Household Survey and analyzed using multiple binary logistic regressions. The analytical sample included 2,653 men, 18 years and older with a chronic illness. Results: Health information seeking was not associated with confidence to manage chronic illnesses. African-American men had lower odds than White men to agree to take actions to prevent symptoms with their health. Hispanic men had lower odds than White men to agree to tell a doctor concerns they have, even when not asked.

**Conclusions:**

Racial and ethnic minority men with a chronic condition appear to be less confident to manage their health compared to white men. Chronic disease management needs greater exploration to understand the best ways to help racial and ethnic minority men successfully manage their chronic condition.

## 1. Introduction

Chronic diseases are prominently featured in the health disparities landscape in the United States among men. Significant health disparities related to mortality and morbidity exists between racial and ethnic minority men (African American and Hispanic) and White men in the United States. Men who belong to racial and ethnic minority populations have elevated rates of chronic disease mortality, disability, and morbidity in comparison to White men [1]–[3]. African-American men have a higher incidence rate of lung, colorectal and prostate cancer compared to White men [4]–[6]. Additionally, Hispanic and African-American men are subject to higher rates of heart disease and diabetes related mortality than White men [7],[8]. Furthermore, racial and ethnic minority men have an earlier onset of chronic diseawhich often presents adverse clinical course with other health complications compared to White men [9]–[12]. Thus, effective self-management of chronic diseases, which encompasses personal involvement in the control of a health condition, is important to improve health outcomes [13].

Studies of men with chronic diseases support the claim that self-confidence in one's ability to manage his disease is pivotal to positive health outcomes [14]. Personal confidence to manage health is a robust predictor of positive behavioral persistence [15],[16]. Its importance is explicitly seen in self-management tasks of chronic conditions, which include medical management of the conditions, maintaining important life roles, and managing negative emotions, such as depression [17]. Furthermore, patients who are more informed have better health outcomes [18].

Health information seeking is associated with patient empowerment, positive health management, patient follow up, and patient treatment decision making [19],[20]. Rooks et al. found health information seeking was associated with changing the approach to managing health and better understanding of how to treat illness [21]. Aslo, importantly, studies that have examined health information seeking found African American and Hispanics are less likely than Whites to use health information to advocate during their medical visit [22],[23]. However, no study has examined the relationship across race/ethnicity of men who seek health information and their confidence to manage their chronic condition, given the noted benefits of health information seeking. The goal of this study was to examine the relationship between health information seeking and confidence in managing chronic conditions and health care interactions among men using nationally-representative data from the Health Tracking Household Survey.

## 2. Materials and Methods

### 2.1. Data and Sampling

Study data were drawn from the 2007 Health Tracking Household Survey (HTHS), a nationally representative, telephone-administered survey of civilian, non-institutionalized individuals. This cross-sectional survey is conducted by the Center for Studying Health System Changes [24]. The HTHS collected information on health and health care markets on randomly selected households across the United States. Detailed descriptions of the HTHS and survey design have been published elsewhere [25],[26]. The analytical sample (aged 18 to 65) included 2,653 men who had at least one medical encounter with a physician in the previous 12 months and have seen a doctor for diabetes, asthma, arthritis, chronic obstructive pulmonary disease, hypertension, coronary health disease, skin or other cancer, benign prostate disease, or depression during the past two years, per HTHS. Male respondents were excluded because of the following: 1) inapplicable, not ascertained, don't know, and refused responses on outcome variables; 2) the race category “Other” was omitted from analysis plan; and 3) missing values on “usual source of care” variable.

Aday and Andersen's Behavioral Model of Health Services Use is the theoretical model, which guides this study [27]. The model suggests health care utilization is a function of predisposing, enabling, and need variables [27]. Predisposing characteristics pre-date the onset of illness for which health care services are sought (e.g., race/ethnicity, education, marital status, and employment status); enabling factors are resources that facilitate or hinder the use of desired or needed health services (e.g., health insurance status, usual source of care, residence, and poverty level); and the need factor is the illness/condition which health care services are sought (e.g., perceived health status). Andersen's models have been used to assess the impact of health information seeking on health behavior [21]–[23].

### 2.2. Variables Under Observation

#### 2.2.1. Dependent variables

The dependent variables are preventing symptoms, knowing when to seek medical care, telling doctors concerns, and following through on medical treatments at home. In the HTHS, respondents were asked if they strongly disagreed, disagreed, agreed, or strongly agreed with the following statements: “I am confident that I can take actions that will help prevent or minimize some symptoms or problems associated with my health (preventing symptoms); I am confident that I can tell when I need to go get medical care and when I can handle a health problem myself (knowing when to seek medical care); I am confident I can tell a doctor concerns I have, even when he or she does not ask (telling doctors concerns); and I am confident I can follow through on medical treatments I need at home (following through on medical treatments at home).”

#### 2.2.2. Independent variables

The main independent variables are health information sought and race. Health information seeking is a dichotomous variable (yes/no) and race is White, African American, or Hispanic. This study defines health information sought as getting health information about a personal health concern from the internet, friends or relatives, television or radio, books or magazines, or newspapers during the past 12 months. Variables representing predisposing, enabling, and need characteristics were included as control variables. The predisposing variables are age (18–24, 25–34, 35–44, 45–54, 55–64, 65 and older), race (White, African American, and Hispanic), marital status (married/not married), and employment status (yes/no). The enabling variables include: rural residence (yes/no); education (< high school, high school, some college, college degree and above); federal poverty level poor (0% to 99%), near poor (100% to 199%), non-poor (200% and up); usual source of care yes/no); and insurance type (private, public, uninsured). The need variable is perceived health status (poor/fair, good, very good/excellent).

#### 2.2.3. Statistical Analysis

Descriptive and logistic regression analyses were performed using STATA 12.28 The sample was weighted to represent the total United States population and to adjust for over-sampling and non-response. Descriptive analyses were conducted to assess variables across racial/ethnic groups. The reported responses to the dependent variables of interest were coded on a 4-point scale in the survey (strongly disagree, disagree, agree, and strongly agree). We collapsed strongly agree and agree into one category (1 = agree) and strongly disagree and disagree into another category (0 = disagree). Logistic regressions (4 total, one for each dependent variable) modeled the odds of agreeing to feel confident in each outcome as a function of the independent variables. Odds ratios (OR) and 95% confidence intervals are presented for each regression model adjusted for each independent variable with goodness of fit assessed by the Hosmer-Lemeshow test [29],[30]. All results were considered statistically significant at *p-value* < 0.05.

## 3. Results

### 3.1. Characteristics of the Study Sample by Race/ethnicity

Table 1 presents the overall weighted percentages for the variables used in this analysis and the weighted percentages for the variables by race/ethnicity. The sample was 82% White, 10% African American, and 8% Hispanic. Over 80% of the men were between the ages of 35–64, 75.8% were married, 61.6% were employed, 92.5% had a usual source of care, 77.2% were non-poor, and 45.4 % were privately insured. Approximately 43.4% of African Americans were married, compared to 74.6% for Whites, and 73.7% for Hispanics. Hispanics were most likely to have less than a high school education (29%), and be uninsured (15.8%), while Whites were the least likely to have less than a high school education (8.9%), be uninsured (4.4%), be poor (7.5%), and be in poor/fair health (26.1%). African-American men were most likely to be unemployed (44.1%) and poor (16%). Overall, 40.2% of the men in this study sought health information from the internet, friends, family, TV or radio, books/magazine/other source.

**Table 1.** Demographics of Participants by Race and Ethnicity.

	Total		White		African American		Hispanic	
	N = 2653	%	N = 2185	%	N = 257	%	N = 211	%
Age								
18–24	78	2.9	57	2.6	15	5.9	7	3.5
25–34	111	4.2	85	3.9	14	5.5	11	5.3
35–44	220	8.3	164	7.5	25	9.8	44	21.0
45–54	488	18.4	376	17.2	73	28.5	39	18.4
55–64	756	28.5	640	29.3	59	22.6	57	27.29
65+	1000	37.7	863	39.5	71	27.7	53	24.6
Married								
No	642	24.2	1709	25.4	145	56.4	156	26.3
Yes	2011	75.8	476	74.6	112	43.4	55	73.7
Rural Living								
Yes	634	23.9	555	24.7	42	16.4	28	13.2
No	5020	76.1	1630	75.3	215	83.6	183	86.8
Educational Level								
< high school	279	10.5	195	8.9	41	16.0	61	29.0
high school	857	32.3	690	31.6	94	36.7	75	35.1
some college	618	23.3	500	22.9	71	27.7	44	21.0
college/post graduate	899	33.9	800	36.6	51	19.5	31	14.9
Employed								
Yes	1634	61.6	1361	62.3	143	55.9	129	61.2
No	1019	38.4	824	37.7	114	44.1	82	38.8
Federal Poverty Level								
Poor (0% to 99%)	231	8.7	164	7.5	41	16.0	34	15.8
Near Poor (100% to 199%)	374	14.1	273	12.5	54	21.1	57	27.2
Non-poor (200%+)	2048	77.2	1748	80.0	162	62.9	120	57.0
Usual Source of Care								
No	199	7.5	146	6.7	27	10.5	33	15.7
Yes	2454	92.5	2039	93.3	230	89.5	178	84.2
Insurance Type								
Private	1205	45.4	1013	46.4	104	40.6	81	38.6
Public	1299	49.0	1075	49.2	125	48.4	97	45.6
Uninsured	149	5.6	97	4.4	28	11.0	33	15.8
Perceived Health Status								
Poor/Fair	751	28.3	570	26.1	104	40.6	92	43.8
Good	912	34.4	761	34.8	81	31.6	67	31.6
Very good/excellent	990	37.3	854	39.1	72	27.7	52	24.6
Sought Health Care								
Yes	1067	40.2	881	40.3	100	38.7	91	43.0
No	1586	59.8	1304	59.7	157	61.3	120	57.0

### 3.2. Confident I Can Take Actions to Help Prevent Symptoms with My Health

Table 2 shows the results from the binary logistic regression examining factors associated with confidence to take actions to help prevent symptoms. African-American men were about 33% less likely than White men to agree they are confident they can take actions to help prevent symptoms with their health (OR: 0.72, CI: 0.53–0.97). Married men were 70% more likely than unmarried men to agree they are confident they can take actions to prevent symptoms (OR: 1.70, CI: 1.14–2.52). Men living in rural areas were 70% more likely than those living in cities to agree they are confident they can take actions to prevent symptoms (OR: 1.70, CI: 1.10–2.88). Men with some college and a college/post graduate degrees were 78% and 130% more likely than those with less than a high school education to agree they are confident they can take actions to prevent symptoms (OR: 1.78, CI: 1.00–3.35 and OR: 2.30, CI: 1.20–4.60). Those who reported good and very good/excellent health were 70% and 207% more likely than those who reported fair/poor health to agree they are confident they can take actions to prevent symptoms (OR: 1.70, CI: 1.08–2.60 and OR: 3.07, CI: 1.90–5.40).

**Table 2.** Odds Ratio of Participants' Confidence to Take Actions to Prevent Symptoms with Their Health.

Characteristics	Odds Ratio	95% CI	*P-value*
Sought Health Information			
No (Reference)			
Yes	1.37	(0.94–1.88)	0.097
Race			
White (Reference)			
African American	0.67	(0.42–0.90)	0.047
Hispanic	1.50	(0.58–4.01)	0.34
Married			
No (Reference)			
Reference	1.70	(1.14–2.52)	0.009
Education Level			
<high school (Reference)			
High school	1.26	(0.75–2.05)	0.378
Some college	1.78	(1.00–3.35)	0.047
College/post graduate	2.30	(1.20–4.60)	0.008
Poverty Level			0.330
Poor (Reference)			0.350
Near poor	0.70	(0.36–1.30)	
Non poor	0.74	(0.35–1.40)	
Rural			0.018
No (Reference)			
Yes	1.78	(1.10–2.88)	
Insurance Type			
Public (Reference)			
Private	0.82	(0.54–1.20)	0.290
Uninsured	0.51	(0.25–1.10)	0.075
Usual Source of Care			
No (Reference)			
Yes	0.74	(0.40–1.66)	0.389
Age	1.00	(0.99–1.00)	0.988
Health Status			
Poor/fair (Reference)			
Good	1.70	(1.08–2.60)	0.030
Very good/excellent	3.07	(1.90–5.40)	0.000

### 3.3. Confident I Know When to Get Medical Care and When I Can Handle Myself

Table 3 lists the results from binary logistic regression on assessing predictors of confidence to know when to get medical care and when I can handle myself. Married men are 50% more likely than unmarried men to agree they are confident in knowing when to get medical care (OR: 1.47, CI: 1.04–2.08). Uninsured men are 56% less likely than men with public insurance to agree they are confident in knowing when to get medical care (OR: 0.44, CI: 0.25–0.77). Men in good and very good/excellent health were 1.57 and 2.11 times more likely to agree they are confident in knowing when to get medical care than those in poor health (OR: 1.57, CI: 1.07–2.29 and OR: 2.11, CI: 1.39–3.18).

**Table 3.** Odds Ratio of Participants' Confidence to Know When to Get Medical Care and Can Handle Themselves.

Characteristics	Odds Ratio	95% CI	*P-value*
Sought Health Information			
No (Reference)			
Yes	0.76	(0.55–1.07)	0.120
Race			
White (Reference)			
African American	1.14	(0.69–1.89)	0.584
Hispanic	0.80	(0.42–1.51)	0.502
Married			
No (Reference)			
Yes	1.47	(1.04–2.08)	0.027
Education Level			
<high school (Reference)			
High school	1.07	(0.64–1.76)	0.789
Some college	1.03	(0.60–1.79)	0.896
College/post graduate	1.67	(0.93–3.00)	0.081
Poverty Level			0.130
Poor (Reference)			0.230
Near poor	1.55	(0.87–2.75)	
Non poor	1.35	(0.82–2.23)	
Rural			0.055
No (Reference)			
Yes	1.47	(0.99–2.18)	
Insurance Type			
Public (Reference)			
Private	1.05	(0.69–1.60)	0.788
Uninsured	0.44	(0.25–0.77)	0.005
Usual Source of Care			
No (Reference)			
Yes	1.28	(0.76–2.15)	0.335
Age	1.00	(0.99–1.01)	0.827
Health Status			
Poor/fair (Reference)			
Good	1.57	(1.07–2.29)	0.019
Very good/excellent	2.11	(1.39–3.18)	0.000

### 3.4. Confident I Can Tell a Doctor Concerns I Have, Even When He or She Does Not Ask

Table 4 presents the results of the model used to explore the determinants of confidence to tell a doctor concerns, even when the doctor does not ask. Hispanic men were 0.50 times less likely to agree that they are confident in telling a doctor concerns compared to White men (OR: 0.50, CI: 0.25–0.99). Non-poor men were 1.78 times more likely to agree that they are confident in telling a doctor concerns compared to those who are poor (OR: 1.78, CI: 1.01–3.15). Those who are uninsured were 0.59 times less likely to agree that they are confident in telling a doctor concerns compared to those who have public insurance (OR: 0.41, CI: 0.21–0.78). Men who reported very good/excellent health were 1.92 times more likely to agree that they are confident in telling a doctor concerns compared to those who are in poor/fair health (OR: 1.92, CI: 1.13–3.24).

**Table 4.** Odds Ratio of Participants' Confidence to Tell a Doctor Concerns, Even When He or She Does Not Ask.

Characteristics	Odds Ratio	95% CI	*P-value*
Sought Health Information			
No (Reference)			
Yes	0.67	(0.44–1.02)	0.063
Race			
White (Reference)			
African American	0.75	(0.43–1.30)	0.844
Hispanic	0.50	(0.25–0.99)	0.045
Married			
No (Reference)			
Yes	1.27	(0.83–1.95)	0.263
Education Level			
< high school (Reference)			
High school	0.82	(0.45–1.49)	0.521
Some college	1.03	(0.52–2.03)	0.932
College/post graduate	1.59	(0.75–3.34)	0.218
Poverty Level			0.222
Poor (Reference)			0.045
Near poor	1.47	(0.79–2.72)	
Non poor	1.78	(1.01–3.15)	
Rural			0.555
No (Reference)			
Yes	0.87	(0.56–1.36)	
Insurance Type			
Public (Reference)			
Private	0.84	(0.50–1.41)	0.533
Uninsured	0.41	(0.21–0.78)	0.008
Usual source of care			
No (Reference)			
Yes	1.22	(0.66–2.26)	0.515
Age	1.01	(0.99–1.02)	0.170
Health Status			
Poor/fair (Reference)			
Good	1.29	(0.81–2.04)	0.274
Very good/excellent	1.92	(1.13–3.24)	0.015

### 3.5. Confident I Can Follow Through on Medical Treatments I Need at Home

Table 5 displays the results for the model to examine the predictors of confidence I can follow through on medical treatments I need at home. Men who report very good/excellent health were 99% more likely than men who report poor/fair health to agree that they are confident they can follow through on medical treatments needed at home (OR: 1.99, CI: 1.11–3.57).

**Table 5.** Odds Ratio of Participants' Confidence to Follow Through on Medical Treatments Needed at Home.

Characteristics	Odds Ratio	95% CI	*P-value*
Sought Health Information			
No (Reference)			
Yes	0.68	(0.43–1.09)	0.112
Race			
White (Reference)			
African American	0.70	(0.38–1.29)	0.258
Hispanic	1.70	(0.51–5.66)	0.385
Married			
No (Reference)			
Yes	1.27	(0.78–2.07)	0.319
Education Level			
< high school (Reference)			
High school	1.10	(0.58–2.08)	0.767
Some college	1.99	(0.90–4.36)	0.086
College/post graduate	1.60	(0.74–3.36)	0.227
Poverty Level			0.882
Poor (Reference)			0.643
Near poor	1.05	(0.50–2.22)	
Non poor	1.17	(0.58–2.35)	
Rural			0.672
No (Reference)			
Yes	0.89	(0.54–1.47)	
Insurance Type			
Public (Reference)			
Private	0.66	(0.37–1.17)	0.163
Uninsured	0.81	(0.33–2.02)	0.663
Usual Source of Care			
No (Reference)			
Yes	1.56	(0.79–3.08)	0.194
Age	1.00	(0.99–1.02)	0.435
Health Status			
Poor/fair (Reference)			
Good	1.39	(0.83–2.34)	0.205
Very good/excellent	1.99	(1.11–3.57)	0.020

## 4. Discussion

This study examined health information seeking and confidence in managing chronic condition and health care interactions among a nationally representative sample of men. Health information seeking had a marginally significant association with confidence to prevent symptoms with health and telling doctors concerns even when not asked. These finding do not parallel those of Rooks et al. who found health information had a significant association with managing health [21]. However, that study did not conduct gender specific analyses. The marginal significance might be attributable to the complexity of chronic diseases. Men might fall into the category of complex patients. Safford et al. contend that patient complexity is a function of the interactions between biological, cultural, environmental, socioeconomic, and behavioral forces [31].

Race was a significant predictor for men agreeing with confidence to manage chronic condition and health care interactions. African-American men had lower odds of agreement to having confidence in taking actions to prevent symptoms compared to White men. The findings [32] are somewhat unexpected due to the high self-reliant behavior and isolation of African-American men. This leaning toward isolation places a greater burden on African-American men to be able to solve their own health problems [33], which might explain their overall poor management of chronic diseases. We also found that Hispanic men had lower odds of agreement to being confident to tell a doctor concerns even when the doctor does not ask compared to White men. This is consistent with Elder et al. who found racial and ethnic minority men were less likely than White men to self-advocate during the medical encounter [23]. Furthermore, racial and ethnic minorities have less participatory visits during the medical encounter compared to White men [34].

There were other predictors of confidence with chronic conditions. Marriage was a positive predictor for agreement in being confident in taking actions to prevent symptoms and knowing when to seek health care for those with a chronic condition. Marriage might be a proxy for social support for men. The literature is replete [35]–[42] with reports of an association between social support and health Social support is positively associated with physical health [35]–[38],[41]–[43], as well as health-related quality of life [44]. Another finding was that rural male residents had higher odds for agreement in being confident in taking actions to prevent symptoms and knowing when to seek health care compared to non-rural residents. Even though rural residents appear somewhat disadvantaged concerning some health metrics, they fare better or no worse than urban residents in health care outcomes [45]. Hartley et al. found rural residents were less likely to have delayed care when they thought care was necessary compared to urban residents [46]. Lastly, those who were uninsured had lower odds for agreement in being confident in taking actions to prevent symptoms and knowing when to seek health care and tell concerns to a doctor even when the doctor does not ask compared to those with public insurance. The uninsured tend to have lower health literacy, less participatory health visits and continuity of care, which might explain the lower confidence in the aforementioned areas [47],[48].

This study has some limitations. Chronic condition type was not specified and length of time the patient had the chronic condition was not reported, both of which might affect confidence to manage health. Health literacy was not included in this study and the findings might be influenced by this measure [49]. Even though the sample does include educational level of the participants, educational level is not equivalent to functional health literacy [50] and minorities have lower health literacy skills compared to Whites [51],[52]. Likewise, the survey did not capture language proficiency or birthplace for Hispanics. This might impact Hispanic men's confidence to manage health since Hispanics are more likely to be influenced by limited English proficiency in health care [53]. This study also did not include an examination of self-efficacy, which is associated with medical adherence in African-American men [54].

## 5. Conclusion

This study found race was a predictor for having confidence to manage health and health care interactions for males with chronic conditions. African-American men had lower odds of agreement to having confidence in taking actions to prevent symptoms than White men. We also found Hispanic men had lower odds of agreement to being confident tell a doctor concerns even when the doctor does not ask compared to White men. Since racial and ethnic minority men are less likely than other groups to seek health care even when they have health insurance [55], the health care encounter should be maximized. The health care system should make certain African-American and Hispanic men understand fully how to manage their chronic conditions and interact with providers successfully. For example, African-American and Hispanic men may benefit from physicians and nurses spending more time with them to build rapport, shared responsibility, and trust. The literature demonstrates that positive physician- patient partnership is associated with better health outcomes and medical adherence [56],[57]. Bodenheimer et al. have suggested primary care physicians could learn about resources available to patients outside of the medical encounter that could help with self-management of health and advocate to health insurance companies to support [58]. Aslo importantly, for patients who are unmarried with no social support, providers could give referals to community support groups or support classes since support is integral for long term health maintenance and promotion in minority patients [58]–[60]. The health care encounter for racial and ethnic minority men should be improved if we hope to change the poor health outcomes associated with chronic conditions for a group that is largely disconnected from the health care system due to health system and personal [48],[61].
